# The Late Osteoblast/Preosteocyte Cell Line MLO-A5 Displays Mesenchymal Lineage Plasticity *In Vitro* and *In Vivo*

**DOI:** 10.1155/2019/9838167

**Published:** 2019-01-17

**Authors:** Dongqing Yang, Stan Gronthos, Sandra Isenmann, Howard A. Morris, Gerald J. Atkins

**Affiliations:** ^1^Biomedical Orthopaedic Research Group, Centre for Orthopaedic and Trauma Research, Adelaide Medical School, Faculty of Health and Medical Sciences, University of Adelaide, Adelaide, SA 5005, Australia; ^2^Mesenchymal Stem Cell Laboratory, Adelaide Medical School, Faculty of Health and Medical Sciences, Adelaide University, Adelaide, SA 5005, Australia; ^3^South Australian Health and Medical Research Institute, Adelaide, SA 5000, Australia; ^4^Musculoskeletal Biology Research, School of Pharmacy and Medical Sciences, University of South Australia, Adelaide, SA 5000, Australia

## Abstract

The process of osteoblast switching to alternative mesenchymal phenotypes is incompletely understood. In this study, we tested the ability of the osteoblast/preosteocyte osteogenic cell line, MLO-A5, to also differentiate into either adipocytes or chondrocytes. MLO-A5 cells expressed a subset of skeletal stem cell markers, including Sca-1, CD44, CD73, CD146, and CD166. Confluent cultures of cells underwent differentiation within 3 days upon the addition of osteogenic medium. The same cultures were capable of undergoing adipogenic and chondrogenic differentiation under lineage-appropriate culture conditions, evidenced by lineage-specific gene expression analysis by real-time reverse-transcription-PCR, and by Oil Red O and alcian blue (pH 2.5) staining, respectively. Subcutaneous implantation of MLO-A5 cells in a gel foam into NOD SCID mice resulted in a woven bone-like structure containing embedded osteocytes and regions of cartilage-like tissue, which stained positive with both alcian blue (pH 2.5) and safranin O. Together, our findings show that MLO-A5 cells, despite being a strongly osteogenic cell line, exhibit characteristics of skeletal stem cells and display mesenchymal lineage plasticity *in vitro* and *in vivo*. These unique characteristics suggest that this cell line is a useful model with which to study aging and disease-related changes to the mesenchymal lineage composition of bone.

## 1. Introduction

Osteoblasts derive from mesenchymal skeletal stem cells (SSCs) and actively lay down the organic phase of bone matrix and regulate its mineralisation. The differentiating osteoblast is commonly thought to have three possible fates, including apoptosis or differentiation into either bone lining cells or mature osteocytes. Osteoblasts, rather than being terminally differentiated, have been shown to retain mesenchymal lineage plasticity [1] and have been shown capable of differentiating into adipocytes and chondrocytes [2–6]. Recently, in mice, it was found that even apparently mature osteocytes, expressing green fluorescent protein (GFP) under control of the dentin matrix protein-1 (Dmp1) promoter, can escape from the bone matrix ex vivo, and in doing so revert to a more immature osteoblastic phenotype [7]. The phenomenon of lineage plasticity among osteoblast lineage cells is of increasing interest with the emerging knowledge surrounding the important regulatory role of the osteocyte in bone homeostasis. Furthermore, the observation that the tightly regulated process of bone formation (osteogenesis) decreases with age concomitant with an increase in bone marrow fat accumulation warrants the characterisation of cell line models permissive for the study of both cell fates.

The cell line MLO-A5 [8] was established from osteoblasts derived from the long bones of transgenic mice expressing SV40 large T antigen under control of a 2.6 kb osteocalcin promoter [9]. Since its isolation in 2001, multiple studies have demonstrated that this is a useful cell model, with which to study the differentiation of late-stage osteoblasts [10], the process of biomineralisation [11, 12], and the transition of osteoblasts to osteocytes [13]. MLO-A5 was initially described as an osteoblastic model that mineralises its extracellular matrix in culture [8], with a pattern of mineralisation indistinguishable from that found in lamellar bone [11]. MLO-A5 has been characterised as a late-osteoblast/preosteocyte model because of the high level of the mineralisation phase marker, tissue nonspecific alkaline phosphatase (*Tnap*) and the preosteocyte marker *E11*/*gp38*, but very low levels of the more mature osteocyte genes *Sost* and *Fgf23* [14].

In order to test whether this osteogenic cell line also exhibited lineage plasticity, we first examined the immunophenotype of these cells and found they expressed markers in common with SSCs. We found that under the appropriate culture conditions, MLO-A5 cells poised to form a bone-like mineralised matrix can rapidly switch fates and undergo adipogenesis and chondrogenesis when cultured under the appropriate conditions *in vitro*. Furthermore, ectopic bone formation assays *in vivo* demonstrated that these cells could form calcified cell masses with areas resembling a woven bone matrix containing embedded osteocyte-like cells and pockets of cartilage. This cell line model thus provides the potential of investigating the cellular and molecular basis of the lineage plasticity of mature osteoblast-like cells.

## 2. Materials and Methods

### 2.1. Cell Culture and Osteogenic Differentiation

MLO-A5 cells were maintained in *α*-MEM media containing 10% FCS, 100 U/ml penicillin, 100 mg/ml streptomycin, 2 mM L-glutamine, and 10 mM HEPES (growth medium), as described previously [15]. For osteogenic differentiation, growth medium was supplemented with *β*-glycerol phosphate (10 mM; Sigma, Australia), dexamethasone (10 nM), and ascorbic acid (50 *μ*g/ml; Sigma Chemical Company, St. Louis, MO, USA) [15]. For differentiation experiments, 3 × 10^4^ cells/well were seeded into 24-well plates in growth medium and allowed to proliferate for 3 days to achieve 100% confluence to a “predifferentiation” stage, and this time point was recorded as differentiation day 0. From day 0, fresh osteogenic differentiation media were supplied to cultures every three days until day 12. Total RNA was extracted from duplicate wells on days 3, 6, and 12, as described below. To quantify mineral deposition at these same time points, triplicate wells were stained using the alizarin red technique, as described previously [16].

### 2.2. Cell Surface Marker Staining and Flow Cytometry Analysis

Mouse primary bone marrow stromal cells (BMSCs) were isolated as described previously [17]. MLO-A5 cells and BMSCs were subjected to immunofluorescence and flow cytometry for reactivity with the monoclonal antibodies STRO-1 [18], anti-Sca-1, CD44, CD73, CD105, CD90, CD106, CD146, and CD166.

### 2.3. Adipogenic Differentiation

For adipogenic induction, confluent cultures of MLO-A5 cells grown in growth medium, as above, were switched (day 0) to media also containing media 60 *μ*M indomethacin, 100 nM dexamethasone, and 50 *μ*g/ml ascorbate-2-phosphate [19]. Fresh adipogenic differentiation medium was supplied to cultures every three days till day 21. *In vitro* fat droplet formation was visualised by Oil Red O (Sigma-Aldrich, Australia) staining on days 7, 14, and 21 of culture.

### 2.4. Chondrogenic Differentiation

For chondrogenic assays, MLO-A5 cells were harvested from 100% confluent 75 cm^2^ tissue culture flasks generated in growth medium (day 0) as above. Aliquots of 1 × 10^6^ cells were pelleted by centrifugation at 600 *g* for 5 min. Cell pellets were supplied with chondrogenic medium consisting of DMEM with 0.125% *w*/*v* bovine serum albumin, tissue culture additives (100 unit/ml penicillin and 100 mg/ml streptomycin, 2 mM L-glutamine, and 10 mM HEPES), 10 *μ*M dexamethasone, 50 *μ*g/ml ascorbate-2-phosphate, and 10 ng/ml TGF-*β*1 (R&D Systems, Minneapolis, MN, USA). Fresh chondrogenic medium was supplied to all cultures every two days till day 21. Total RNA was collected on days 3, 6, 12, and 21. On day 12, a cell pellet culture was also fixed in 4% *w*/*v* paraformaldehyde overnight for paraffin embedding and further histological analyses including staining with safranin O and alcian blue (pH 2.5) [19].

### 2.5. Glycosaminoglycan (GAG) Assay

Chondrogenic differentiation was assessed in high-density (0.5-1 × 10^5^ cells per well) cultures in 96-well plates with 10 ng/ml TGF-*β*1 for 48 hours as described previously [19]. Glycosaminoglycan synthesis was measured in quadruplicate wells by ^35^SO_4_ incorporation using a TopCount NXT Microplate Scintillation & Luminescence counter (Perkin Elmer Life and Analytical Sciences, Downers Grove, IL). Values for GAG synthesis were normalised to DNA content per well.

### 2.6. Xenograft Model

Approximately 5.0 × 10^6^ MLO-A5 cells from confluent cultures, as above, were added to a gel-foam carrier (Zimmer Inc., Warsaw, IN) and then transplanted subcutaneously into the dorsal surface of 10-week-old immunocompromised NOD/SCID mice for 10 weeks as previously described [19]. MLO-A5-implanted carriers were fixed in 10% *v*/*v* buffered formalin and decalcified. Safranin O staining and alcian blue staining (with pH 2.5) were performed to characterise osteogenesis and chondrogenesis. An anti-SV40 large T antigen antibody (Santa Cruz Biotechnology, Texas, USA) followed by a peroxidase detection system (Envision, Detection Systems, Peroxidase/DAB, Dako, Glostrup, Denmark) was used to confirm the cell origin within the tumour tissue. These procedures were performed in accordance to specifications of an approved animal protocol (University of Adelaide Animal Ethics Committee number M-2012-207 and SA Pathology Ethics Committee number 141/12).

### 2.7. Gene Expression Analysis

Total RNA was extracted from the cell cultures using the TRIZOL method (Invitrogen, Australia), and cDNA samples were synthesised (Superscript III) to measure the expression of genes of interest in mRNA level by real-time PCR, as described previously [15]. The primer sequences for measuring each gene are listed in Table 1. Gene expression was normalised for the housekeeping gene *β*-actin.

## 3. Results

### 3.1. MLO-A5 Cells Express a MSC-Like Immunophenotype

**Figure 1:**
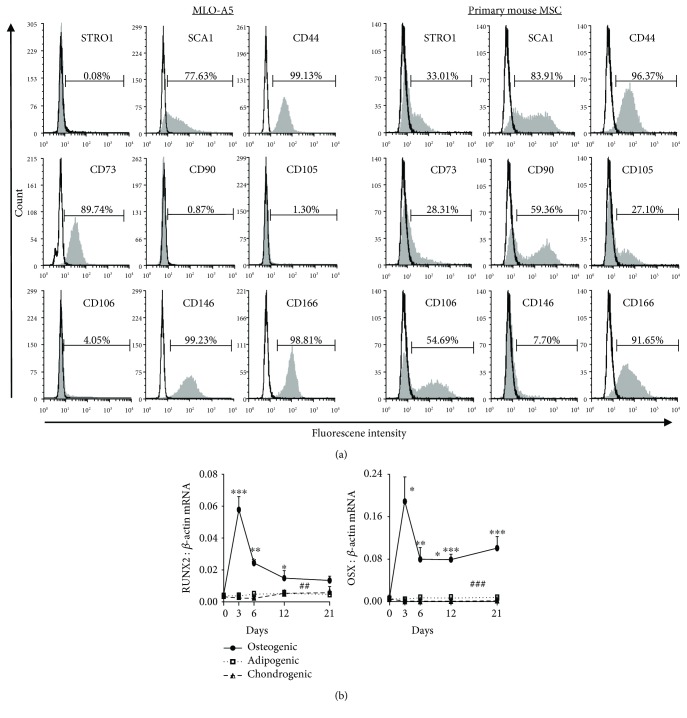
(a) Immunophenotypic analysis of MLO-A5 by flow cytometry compared with mouse primary BMSCs. (b) Relative (normalised to *Actb*) mRNA expression levels of *Runx2* and *Osx* under osteogenic, adipogenic, and chondrogenic conditions. Data shown are means of biological triplicates (*n* = 3) ±SEM; significant differences to day 0 value indicated by ^∗^*p* < 0.05 and ^∗∗∗^*p* < 0.001; significant higher *Runx2* or *Osx* mRNA levels under osteogenic compared to adipogenic or chondrogenic conditions is indicated by ^##^*p* < 0.01 and ^###^*p* < 0.001).

Flow cytometric analyses demonstrated that while the SSC surface markers STRO-1, CD90, CD105, and CD106 were barely detectable in MLO-A5 cells, the SSC markers Sca-1, CD44, CD73, CD146, and CD166 were expressed at high levels (Figure 1(a)). In comparison, mouse BMSCs expressed the expected immunophenotype. This suggested that MLO-A5 cells may possess at least some SSC-like qualities with respect to mesenchymal lineage commitment.

The mRNA levels of osteogenic specific transcriptional factors, runt-related transcription factor 2 (*Runx2*) and osterix (*Osx*) (Figure 1(b)), were on average 20-fold higher in osteogenic cultures compared to the cultures treated with adipogenic or chondrogenic conditions. Notably, both *Runx2* and *Osx* mRNA levels rapidly increased from the day 0 value when cells were cultured in osteogenic media, peaking after 3 days, while the levels of these two genes remained unchanged with culturing under adipogenic or chondrogenic conditions.

### 3.2. Osteogenic Differentiation

**Figure 2:**
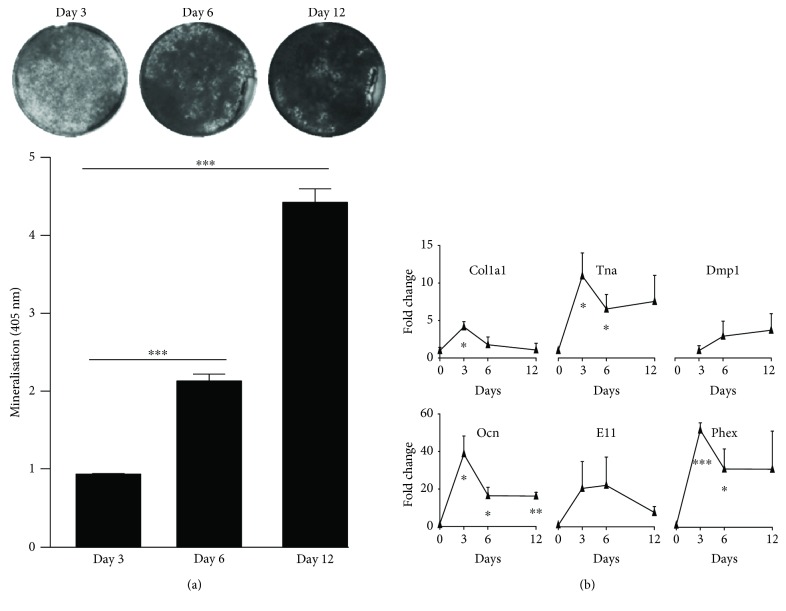
(a) *In vitro* mineral deposition by MLO-A5 determined by Alizarin Red staining and quantification, as described in Materials and Methods. Data shown are means of experimental triplicates (*n* = 3) ± SEM; significant changes in mineralisation level are indicated by ^∗∗∗^*p* < 0.001. (b) Real-time RT-PCR analysis of *Col1a1*, *Tnap*, *Dmp1*, *Ocn, E11*, and *Phex* gene expression in MLO-A5 cultures under osteogenic conditions. Data are shown as mean fold − change ± SEM of biological triplicates assayed in duplicate from the day 0 value for each gene, except for *Dmp1* which was normalised to the day 3 value due to undetectable levels at day 0. Significant changes are indicated by ^∗^*p* < 0.05, ^∗∗^*p* < 0.01, and ^∗∗∗^*p* < 0.001.

Confluent cultures switched to osteogenic inductive conditions and underwent extensive mineralisation after just 3 days (Figure 2(a)), suggesting that by virtue of reaching confluence at day 0, the cells had already reached a predifferentiated osteogenic state. The degree of mineral deposition increased progressively with time evidenced by alizarin red staining (Figure 2(a)). The mRNA levels of osteogenic differentiation-related genes in MLO-A5 cultures, including collagen type I alpha 1 (*Col1a1*), *Tnap*, osteocalcin (*Ocn*), and X-linked phosphate-regulating endopeptidase (*Phex*) (Figure 2(b)), increased significantly by day 3 compared to day 0 levels. The mRNA expression of dentin matrix protein 1 (*Dmp1*) and *E11* did not change significantly over this period.

### 3.3. Adipogenic Differentiation

**Figure 3:**
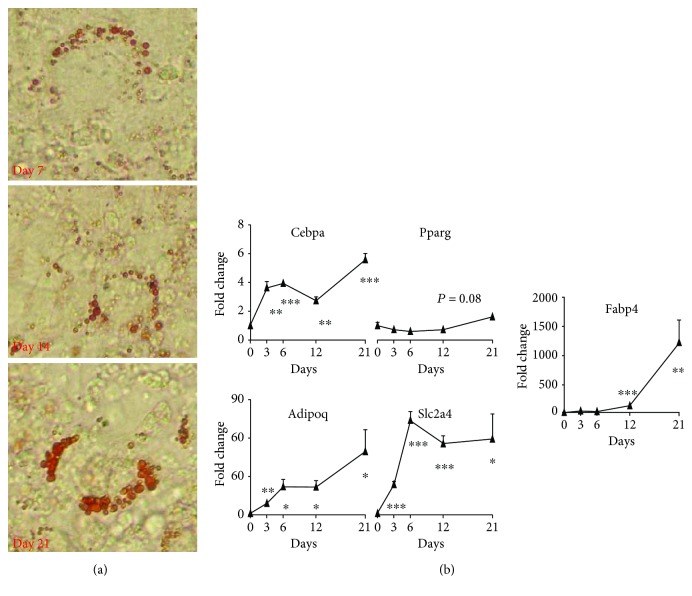
(a) *In vitro* fat droplet formation by MLO-A5 cultured under adipogenic conditions for the times indicated, visualised by Oil Red O staining. (b) Real-time RT-PCR analysis of *Cebpa*, *Pparg*, *Adipoq*, *Slc2a4*, and *Fabp4* gene expression in MLO-A5 cultures under adipogenic conditions. Data are shown as mean fold − change ± SEM of biological triplicates assayed in duplicate from the day 0 value for each gene. Significant changes are indicated by ^∗^*p* < 0.05, ^∗∗^*p* < 0.01, and ^∗∗∗^*p* < 0.001.

Under adipogenic inductive conditions, predifferentiated cultures of MLO-A5 developed oil droplet formation over a period of 7 days, as evidenced by Oil Red O staining (Figure 3(a)). The mRNA levels of adipogenic differentiation-related genes in MLO-A5 cultures including CCAAT/enhancer-binding protein, alpha (*Cebpa*), peroxisome proliferator-activated receptor gamma (*Pparg*), adiponectin (*Adipoq*), solute carrier family 2 (*Slc2a4*), and fatty acid-binding protein 4 (*Fabp4*) all significantly increased compared to day 0 levels (Figure 3(b)).

### 3.4. Chondrogenic Differentiation

**Figure 4:**
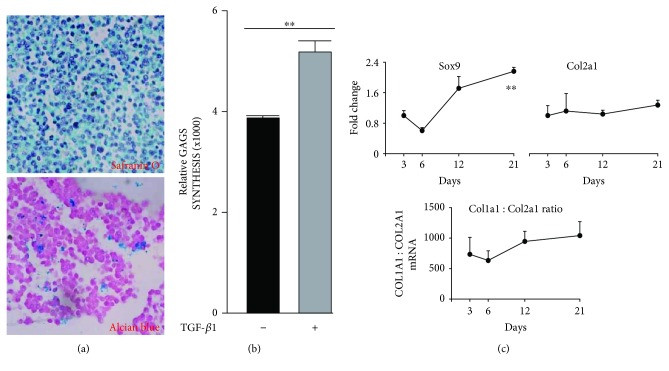
(a) *In vitro* chondrogenesis by MLO-A5 in pellet cultures, as described in Materials and Methods, visualised by safranin O staining and alcian blue staining at pH 2.5. (b) GAG synthesis in MLO-A5 cells cultured under chondrogenic conditions in the presence or absence of recombinant TGF-*β*1. Data shown are means ± SEM of readings from quadruplicate wells; significance is indicated by ^∗∗^*p* < 0.01. (c) Chondrogenic gene expression determined by real-time RT-PCR analysis of *Sox9* and *Col2a1* as well as the *Col1a1*/*Col2a1* ratio. Data are shown as mean fold − change ± SEM of biological triplicates assayed in duplicate from the day 3 value for each gene. Significant changes are indicated by ^∗∗^*p* < 0.01.

MLO-A5 cultures switched to chondrogenic inductive conditions stained negative by the safranin O method but positive under alcian blue staining at pH 2.5, suggesting the formation of unsulphated but not sulphated glycosaminoglycans [20–22] (Figure 4(a)). *In vitro* production of glycosaminoglycan synthesis under chondrogenic conditions was significantly increased by 30% (*p* < 0.01) with the addition of TGF-*β*1 (Figure 4(b)). The mRNA level of the chondrogenic transcriptional factor, sex-determining region Y-box 9 (*Sox9*), increased compared to day 3 level (Figure 4(c)). The mRNA levels of *Col1a1* and collagen type II alpha 1 (*Col2a1*) showed no significant changes over the 21-day culture period. Notably, the levels of *Col1a1* were on average approximately 1500-fold higher than *Col2a1* mRNA levels (Figure 4(c)). The mRNA expression of the chondrocyte matrix-related genes, aggrecan (*Acan*) and collagen type X alpha 1 (*Col10a1*), was undetectable, based on a 45-cycle real-time PCR reaction used in this study (data not shown).

### 3.5. Xenograft Implantation

**Figure 5:**
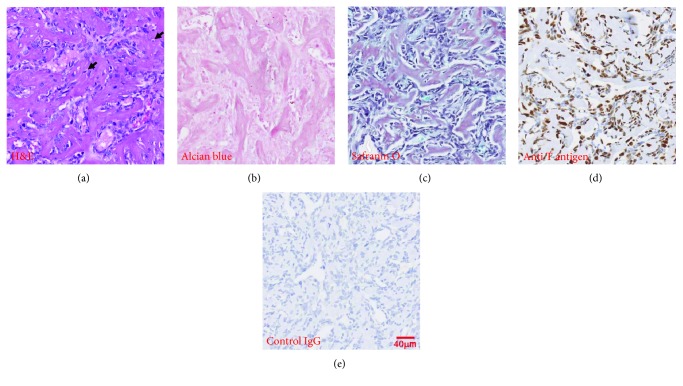
Histological analysis of woven bone-like subcutaneous MLO-A5 tumour grafts: (a) H&E staining, with osteocyte lacunae evident (arrows); (b) alcian blue staining at pH 2.5; (c) safranin O staining; (d) immunostaining of small and large T antigens indicative of MLO-A5 origin; and (e) immunostaining with IgG negative control antibody. All images are of an identical magnification with the scale bar representing 40 *μ*m and are representative of tissue from 3 mice.

After 10 weeks postimplantation of MLO-A5 grafts into NOD SCID mice, an ectopic bone/cartilage organ was observed. Histological analyses (H&E staining) revealed a woven bone-like tissue inside the graft containing embedded osteocyte-like cells (Figure 5(a)). Both alcian blue staining at pH 2.5 (Figure 5(b)) and safranin O staining (Figure 5(c)) confirmed that there was at least some genuine cartilage formation within the tumour graft. Specific immunostaining for the SV40 small and large T antigens confirmed that a large proportion of the cells was of MLO-A5 origin (Figures 5(d) and 5(e)).

## 4. Discussion

The osteoblast cell line MLO-A5 is a useful model, with which to study the process of mineralisation during osteoblast-osteocyte transition under osteogenic culture conditions, and does so in manner entirely consistent with this process *in vivo* [11]. In this study, we explored the mesenchymal lineage plasticity of the late osteoblast, using MLO-A5 cells already cultured to confluence and poised to rapidly become mineralised osteoblasts/preosteocytes [8]. Consistent with previous characterisation [8], “predifferentiated” MLO-A5 cells displayed strong capacity for osteogenic differentiation *in vitro*, evidenced by the rapid and extensive deposition after just 3 days of calcium phosphate mineral in monolayer cultures, and the expression of a panel of osteogenic differentiation-related markers including *Runx2*, *Osx*, *Tnap*, *Ocn*, *E11*, *Phex*, and *Dmp1*. Besides the osteoblastic phenotype, MLO-A5 cells display certain immunophenotypic features of SSC, evidenced by the cell surface expression of Sca-1, CD44, CD73, CD146, and CD166 [17, 23]. However, the minimal criteria for a true multipotent stromal cell include the expression of CD73, as well as CD90 and CD105 [24]. The absence of CD90 and CD105, together with the absence of the markers, STRO-1 and CD106, indicates that this cell line represents a late rather than an early SSC model. Despite this, MLO-A5 retains the capacity to differentiate into adipocyte and chondrocyte lineages, as well as undergoing osteogenesis, which to our knowledge is unique among available cell lines.

By switching these predifferentiated osteogenic cultures to adipogenic media, the formation of oil droplets and the mRNA levels of adipogenic-related genes including *Cebpa*, *Pparg*, *Adipoq*, *Scl2a4*, and *Fabp4* were all induced, consistent with MLO-A5 cells retaining adipogenic lineage plasticity [17]. In chondrogenic cultures, the cartilage matrix products, GAGs, are visualised by alcian blue staining at pH 2.5, with both sulphated and unsulphated forms being detected [21, 22]. In contrast, safranin O staining only detects sulphated GAGs [20]. In this study, chondrogenic pellet cultures of MLO-A5 cells after 21 days did not stain positive by safranin O but were positive by alcian blue. In articular cartilage *in vivo*, unsulphated GAGs are connected by the protein aggrecan, encoded by the *Acan/ACAN* gene, which results in the formation of sulphated GAGs, and which are indicative of mature cartilage matrix [25]. Consistent with the staining pattern observed, the expression of *Acan* mRNA in the MLO-A5 chondrogenic cultures was undetectable after 45 cycles of real-time RT-PCR. Hence, in this study, only unsulphated and possibly nonfunctional GAGs are synthesised by MLO-A5 cells *in vitro*, presumably because of the lack of aggrecan expression. Although the mRNA expression level of the chondrogenic transcription factor, *Sox9*, increased significantly over the 21-day period, the level of the downstream gene *Col2a1* encoding type II collagen, the major collagenous protein in the developing articular cartilage tissue [26], remained unchanged. Our findings demonstrate that MLO-A5 cells are able to undergo a limited degree of adipogenic and chondrogenic differentiation when cultured under the appropriate culture conditions *in vitro*. However, the mechanism of MLO-A5 cell plasticity either via direct or indirect conversion to other lineages requires further assessment. For example, the extent to which cell division is required for lineage switching to occur, or whether a subpopulation of the cells differentiate directly into either adipocytes or chondrocytes, will be important to determine. Future studies will also investigate the transcriptional and epigenetic mechanisms that mediate dedifferentiation and lineage determination. In the xenograft model, where MLO-A5 cells in a gel-foam carrier were implanted subcutaneously into immunodeficient mice, MLO-A5-derived woven bone containing embedded osteocyte-like cells together with cartilage deposits was observed, evidenced by both safranin O and alcian blue staining within the ectopic organ formed, suggesting that additional host factors promote complete chondrogenic differentiation in this model.

## 5. Conclusions

To summarise, we have demonstrated that the MLO-A5 cell line displays mesenchymal plasticity *in vitro* and *in vivo* and is capable of undergoing either adipogenic or chondrogenic differentiation, despite this being a late-osteoblast/preosteocyte, strongly osteogenic cell line. However, the capacity of MLO-A5 cells to undergo adipogenesis and chondrogenesis is incomplete. Whether the lack of the stem cell markers CD90, CD105, and CD106 explains the inability of MLO-A5 to fully differentiate towards adipocytes and chondrocytes is yet to be determined. Furthermore, due to its original method of isolation from transgenic mice, with insertion of the transgene into the genome, and the possibility that the cell line carries unidentified chromosomal abnormalities that may impact on its ability to undergo multilineage differentiation, findings using this cell line should be treated with appropriate caution. Nevertheless, our findings with this cell line suggest that even up to a late stage of osteogenic differentiation, osteoblasts are still capable of switching to an alternative mesenchymal lineage. Recently, methotrexate-based chemotherapy induced bone loss and fat formation was shown to be attributed to the disruption of balance between adipogenic, osteogenic, and osteoclastogenic differentiation within the bone marrow [27]. Therefore, in addition to this cell line being a useful model, with which to study mesenchymal plasticity in the context of a cell line that can form mineralised bone, MLO-A5 is also potentially useful for studying the molecular and cellular basis of the age and disease-associated phenomenon of bone to fat formation.

## Figures and Tables

**Table 1:** Oligonucleotide primer sets used for real-time RT-PCR.

Gene	Sequence (5′-3′)	GenBank accession no.	Amplicon (bp)
*β-Actin*	Forward: cacacccgccaccagtt Reverse: ggaatacagcccgggga	NM_007393.5	115
*Runx2*	Forward: cacaaggacagagtcagattacagat Reverse: cgtggtggagtggatggat	NM_001146038.2	122
*Osx*	Forward: gcgtcctctctgcttgagg Reverse: ggcttctttgtgcctcctttc	NM_130458.3	137
*Col1a1*	Forward: aggcataaagggtcatcgtg Reverse: cgttgagtccgtctttgcca	NM_007742.3	155
*Tnap*	Forward: tcctgaccaaaaacctcaaagg Reverse: tgcttcatgcagagcctgc	NM_007431.2	101
*Dmp1*	Forward: gaaagctctgaagagaggacggg Reverse: tgtccgtgtggtcactatttgcct	NM_016779.2	121
*Ocn*	Forward: agacctagcagacaccatga Reverse: gaaggctttgtcagactcag	NM_010288.3	79
*E11*	Forward: aaacgcagacaacagataagaaagat Reverse: gttctgtttagctctttagggcga	NM_010329.2	158
*Phex*	Forward: gaaaagctgttcccaaaacagag Reverse: tagcaccataactcagggatcg	NM_011077.2	156
*Cebpa*	Forward: ccatgccgggagaactcta Reverse: ctctggaggtgactgctcatc	NM_001287514.1	89
*Pparg*	Forward: gatgcaagggttttttccg Reverse: ccaaacctgatggcattgt	NM_001308352.1	160
*Adipoq*	Forward: tgtccccatgagtaccagact Reverse: cctgagcccttttggtgtc	NM_009605.5	98
*Sla2a4*	Forward: catgtgtggctgtgccatc Reverse: ggcagctgagatctggtcaaac	NM_012751.1	370
*Fabp4*	Forward: ttgtgggaacctggaagct Reverse: ccccatttacgctgatgatc	NM_024406.2	129
*Acan*	Forward: cttctgtgcggctcaaaat Reverse: ccactgacacacctcggaa	NM_007424.2	251
*Col2a1*	Forward: cgagtggaagagcggagactac Reverse: ccagtttttccgagggacagt	NM_001113515.2	136
*Col10a1*	Forward: caatacttcatcccatacgcc Reverse: ctggcacagaaattccagc	NM_009925.4	346
*Sox9*	Forward: cacggaacagactcacatctctc Reverse: tgagattgcccagagtgctc	NM_011448.4	120

## Data Availability

The data used to support the findings of this study are included within the article.

## Abbreviations

*Dmp1*: Dentin matrix protein-1
Tnap: Tissue nonspecific alkaline phosphatase
*E11/gp38*: Podoplanin
*Sost*: Sclerostin
*Fgf23*: Fibroblast growth factor 23
*Runx2*: Runt-related transcription factor 2
*Osx*: Osterix
*Col1a1*: Collagen type I alpha 1
*Ocn*: Osteocalcin
*Phex*: X-linked phosphate-regulating endopeptidase
*Cebpa*: CCAAT/enhancer-binding protein, alpha
*Pparg*: Peroxisome proliferator-activated receptor gamma
*Adipoq*: Adiponectin
*Slc2a4*: Solute carrier family 2
*Fabp4*: Fatty acid-binding protein 4
*Sox9*: Sex-determining region Y-box 9
*Col2a1*: Collagen type II alpha 1
*Col10a1*: Collagen type X alpha 1
*Acan*: Aggrecan
BMSC: Bone marrow stromal cell
SSC: Skeletal stem cell
GAG: Glycosaminoglycan.
